# Predicting Cervical Lymph Node Metastasis Following Endoscopic Surgery in Superficial Head and Neck Carcinoma

**DOI:** 10.3389/fsurg.2021.813260

**Published:** 2022-02-11

**Authors:** Ryuichi Okabe, Yushi Ueki, Riuko Ohashi, Manabu Takeuchi, Satoru Hashimoto, Takeshi Takahashi, Ryusuke Shodo, Keisuke Yamazaki, Hiroshi Matsuyama, Hajime Umezu, Shuji Terai, Yoichi Ajioka, Arata Horii

**Affiliations:** ^1^Department of Otolaryngology-Head and Neck Surgery, Niigata University Graduate School of Medical and Dental Sciences, Niigata, Japan; ^2^Department of Otorhinolaryngology, Nagaoka Red Cross Hospital, Niigata, Japan; ^3^Division of Molecular and Diagnostic Pathology, Niigata University Graduate School of Medical and Dental Sciences, Niigata, Japan; ^4^Histopathology Core Facility, Niigata University Faculty of Medicine, Niigata, Japan; ^5^Division of Gastroenterology and Hepatology, Niigata University Graduate School of Medical and Dental Sciences, Niigata, Japan; ^6^Division of Gastroenterology, Nagaoka Red Cross Hospital, Niigata, Japan; ^7^Department of Otorhinolaryngology, Niigata City General Hospital, Niigata, Japan; ^8^Division of Pathology, Niigata University Medical and Dental Hospital, Niigata, Japan

**Keywords:** cervical lymph node metastasis, endoscopic resection, superficial head and neck carcinoma, classification of type B vessels, tumor thickness

## Abstract

**Background:**

Early detection of head and neck carcinoma (HNC) as superficial HNC (SHNC) identified using recently developed optical techniques, such as magnifying endoscopy and narrow-band imaging (NBI), in combination with endoscopic surgeries enables minimally invasive treatment with favorable outcomes for HNC. This study aimed to identify the predictive factors for the rare but important clinical issue of SHNC, namely cervical lymph node metastasis (CLNM), following endoscopic resection.

**Methods:**

Sixty-nine patients with SHNC who underwent endoscopic resection were enrolled in the study. Clinical data, preoperative endoscopic findings, pathological findings, and treatment outcomes were retrospectively reviewed. Because the pharyngeal mucosa lacks the muscularis mucosa, we measured tumor thickness in permanent pathology as an alternative to the depth of invasion. Correlations with the occurrence of CLNM were statistically examined.

**Results:**

The 5-year disease-specific survival rate was 100%. Of 69 patients, 3 (4.3%) developed CLNM. All had subepithelial but not epithelial tumors. The 0-IIa type in the macroscopic findings, type B2/B3 vessels in narrow-band imaging, tumors ≥ pathological stage T2, lymphatic invasion, positive surgical margins, and tumor thickness >1,000 μm showed significant correlations with CLNM following endoscopic resection. Furthermore, the classification of type B vessels was significantly associated with tumor thickness.

**Conclusion:**

The treatment outcomes following endoscopic resection for SHNC were favorable. The risk of CLNM following endoscopic resection in SHNC can be predicted by several preoperative endoscopic and postoperative pathological findings. Among them, the classification of type B vessels, which correlated with both tumor thickness and CLNM, might be a useful predictive factor.

## Introduction

Head and neck carcinoma (HNC), particularly laryngeal or pharyngeal carcinoma, is often found as an advanced-stage disease (1). Therefore, invasive interventions, including laryngectomy or concurrent chemoradiotherapy, are needed for curative therapy. This usually results in a swallowing disturbance or loss of voice, aggravating the quality of life (1). Developed optical techniques such as narrow-band imaging (NBI) and magnifying endoscopy facilitate the detection of HNC at an early phase, i.e., superficial HNC (SHNC) (2).

**Figure 1:**
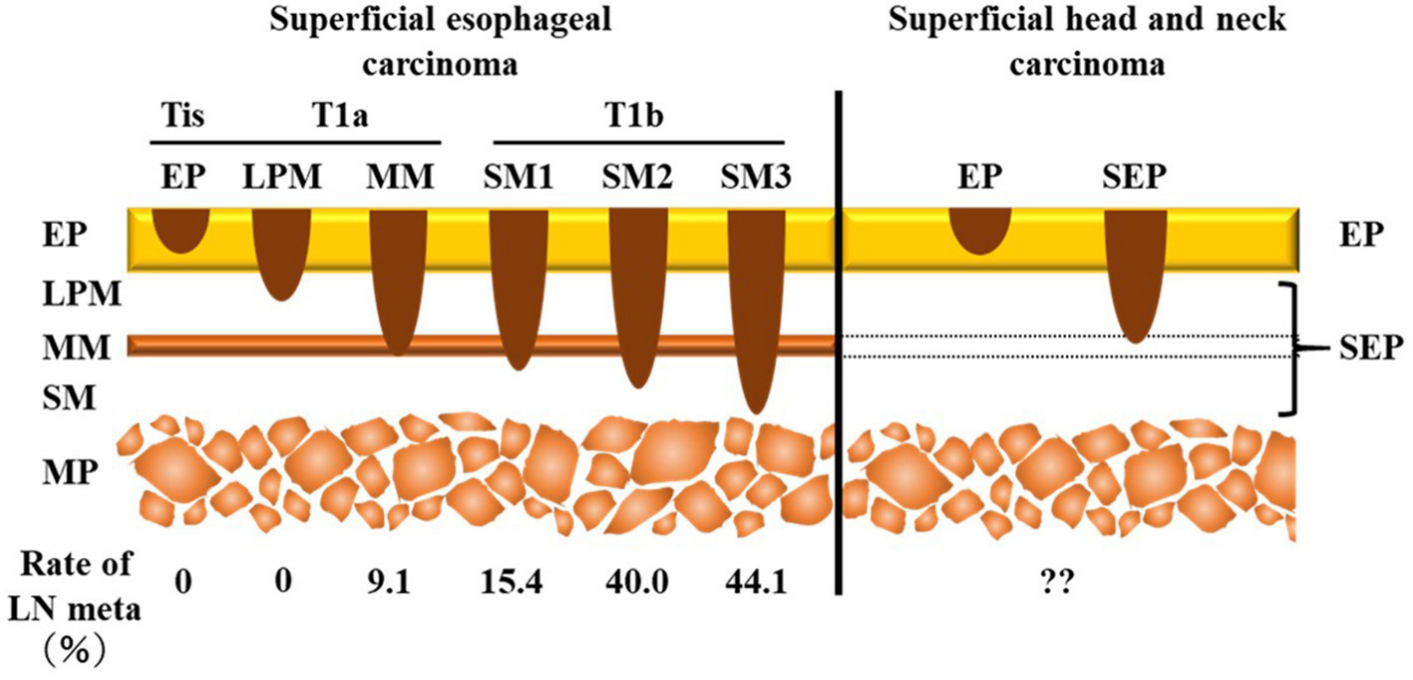
Comparison of the depth of invasion between superficial esophageal carcinoma and superficial head and neck carcinoma. Superficial carcinoma does not invade the muscularis propria. Tis is an *in situ* carcinoma. According to the presence of invasion over the muscularis mucosa, T1 superficial esophageal squamous cell carcinoma is classified into two categories, namely T1a (LPM, MM) and T1b (SM1, SM2, SM3). Superficial head and neck carcinoma is divided into two subgroups—EP (Tis) and SEP tumors—due to the lack of muscularis mucosa. The incidence of lymph node metastasis is different between Tis/T1a and T1b in superficial esophageal carcinoma. LN, lymph node; EP, epithelium; LPM, lamina propria mucosa; MM, muscularis mucosa; SM, submucosa; MP, muscularis propria; SEP, subepithelium.

SHNC is defined as cancer involving the epithelium/subepithelial layer but not the muscularis propria irrespective of the presence of cervical lymph node metastasis (CLNM) (Figure 1) (3–5). The treatment strategy for SHNC follows a plan similar to that of superficial esophageal squamous cell carcinoma (6). Minimally invasive transoral endoscopic resection such as endomucosal resection, endoscopic submucosal dissection, and endoscopic laryngopharyngeal surgery but not chemoradiotherapy or extensive surgery may be a suitable and sufficient treatment for SHNC as well as superficial esophageal squamous cell carcinoma. Indeed, it achieves a favorable outcome and well-maintained postoperative functions (7). However, CLNM can occur in 2–19.4% of SHNC cases (8–12), which, in turn, is a clinical issue in the management of SHNC.

In the treatment of superficial esophageal squamous cell carcinoma, the incidence of lymph node metastasis depends on the depth of tumor invasion: 0–9.1% in Tis (epithelium [EP]) or T1a tumor (lamina propria mucosa [LPM] and muscularis mucosa [MM]) and 15.4–44.1% in T1b tumor (submucosa [SM]1–SM3) (Figure 1) (13). Thus, according to the National Comprehensive Cancer Network guidelines for esophageal carcinoma (14), endoscopic resection is recommended as initial definitive therapy for Tis and T1a superficial esophageal carcinoma, whereas an adjuvant therapy is recommended for T1b esophageal carcinoma due to the high risk of lymph node metastasis. Therefore, an accurate evaluation of the depth of invasion, which separates T1b from Tis/T1a tumor, is essential to determine the treatment strategy for superficial esophageal squamous cell carcinoma. For this purpose, NBI under magnifying endoscopy, which assesses the microvessel irregularity of the tumor surface, is useful to predict the depth of invasion (15).

In contrast to the esophagus, the pharyngeal mucosa lacks the MM, a key structure for determining the depth of invasion that predicts lymph node metastasis in superficial esophageal squamous cell carcinoma (Figure 1). Therefore, SHNC is classified only into two levels, namely EP and subepithelium (SEP) (Figure 1). The Japanese Society for Head and Neck Cancer recommends applying tumor thickness as an alternative to the depth of invasion to assess the risk of vessel invasion (3). Several reports have shed light on the high risk of CLNM for tumors with >1,000 μm thickness (8, 12, 16). However, they did not conduct statistical comparisons with non-CLNM cases. Moreover, previous reports included mixed CLNM cases with both synchronous and metachronous metastases even though the risk factors for metachronous CLNM are more important than those for synchronous CLNM in terms of prognosis. The aims of the present study were to identify the predictive factors for metachronous CLNM following endoscopic resection of SHNC among several preoperative endoscopic and postoperative pathological findings.

## Patients and Methods

### Patients

This study was approved by the appropriate institutional review board (No. 2538) and was conducted in accordance with the ethical standards of the 1964 Declaration of Helsinki and its later amendments. Our retrospective cohort study included 69 patients with SHNC who were treated with endoscopic resection between 2007 and 2017. Patients who had received prior radiotherapy on the head and neck or had cervical lymph node metastasis at the initial diagnosis were excluded from the study. Written informed consent was obtained from all study participants.

### Assessment of Endoscopic Findings

**Figure 2:**
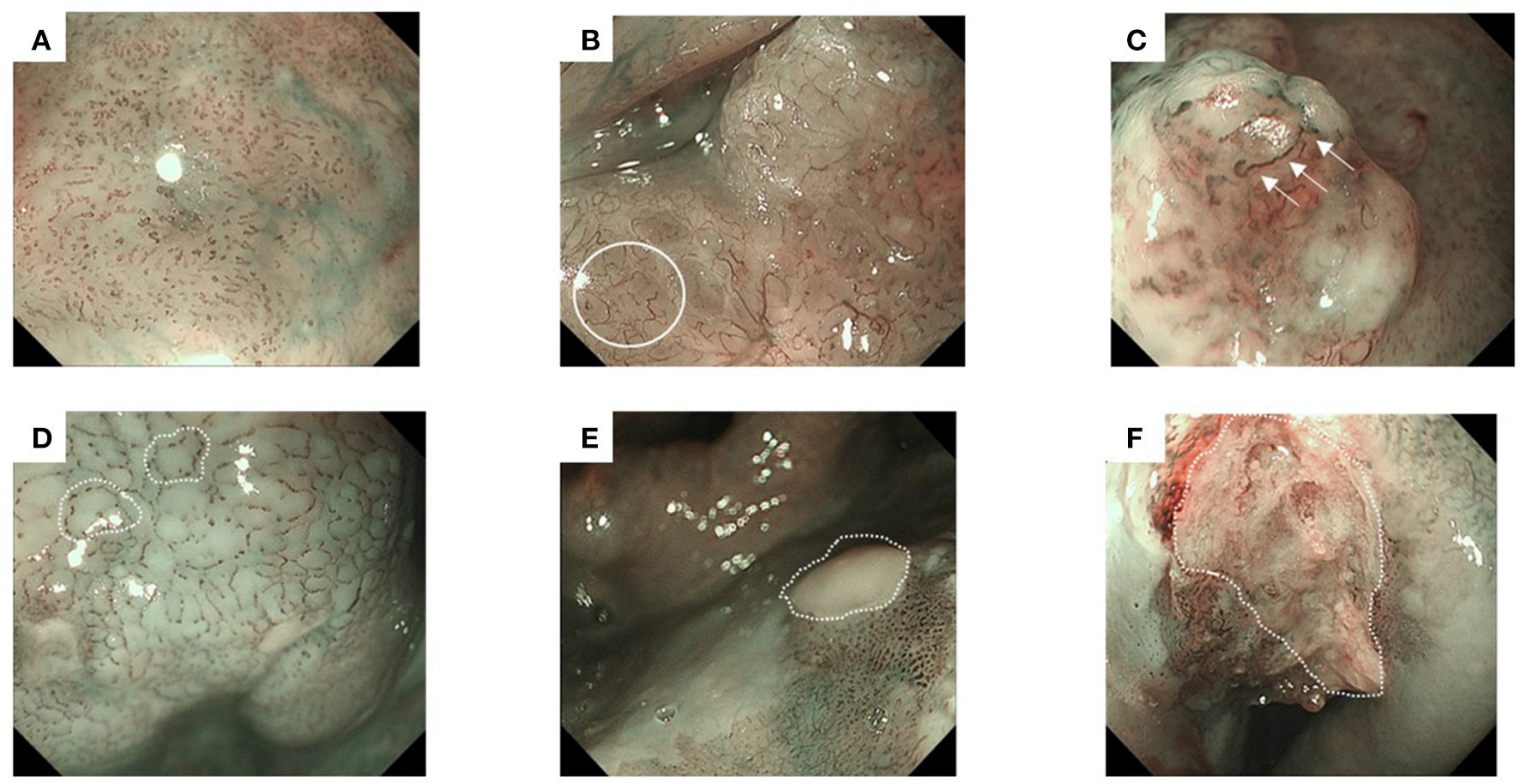
Representative images of superficial head and neck cancer according to the classification of microvessel irregularity under narrow band imaging. Type B vessels show the following four characteristics: weaving, dilation, irregular caliber, and different shapes. They are further subdivided into B1–B3 vessels. Avascular area (AVA) is defined as a low or no vascularity area, surrounded by stretched irregular vessels, such as B2 or B3 vessels. The diameter of AVA is positively correlated with the depth of invasion. Thus, AVA is further categorized according to its diameter. **(A)** Type B1 vessels have a loop-like formation, which appears as dot-like microvessels (e.g., a brownish area). **(B)** Type B2 vessels lack the loop-like formation (white circle). **(C)** Type B3 vessels have highly dilated abnormal vessels (white arrows). **(D)** Small-sized AVA (<0.5 mm in diameter), **(E)** middle-sized AVA (0.5≤, <3mm), and **(F)** large-sized AVA (≥3 mm).

We classified the endoscopic findings of the tumor type and microvessel irregularity according to the Japan Esophageal Society classification (17). Superficial cancer was categorized as type 0, which was further classified into the following stages: 0-I (protruding type), 0-II (flat type), and 0-III (excavated type). Types 0-I and 0-II were further subclassified into 0-Ip (pedunculated type), 0-Is (sessile [broad based] type), 0-IIa (slightly elevated type), 0-IIb (flat type), and 0-IIc (slightly depressed type) (18). Furthermore, microvessel irregularity under NBI was classified into type B1, B2, and B3 vessels (Figures 2A–C). Avascular area (AVA) was defined as a low or no vascularity area surrounded by stretched irregular vessels, such as B2 or B3 vessels (Figures 2D–F) (1).

### Analysis of Predictive Factors

We extracted the clinical data (e.g., age, sex, primary tumor site, synchronous or metachronous carcinoma), treatment outcome (i.e., disease-free survival and overall survival), and endoscopic findings (i.e., macroscopic findings and microvessel irregularity) from the electronic clinical records. A gastroenterologist (SH and MT) evaluated the endoscopic findings (macroscopic type and microvessel irregularity). All histological slides were reviewed by three pathologists (RO, HU, and YA), who were blinded to the clinical data. The pathological T stage was determined according to the 8th edition of the American Joint Committee on Cancer's cancer staging system (19). Additionally, we classified the depth of invasion in SHNC as either EP (tumor located in the epithelium, equivalent to carcinoma *in situ*) or SEP (tumor involving the subepithelial layer). Tumor thickness, defined as the distance from the tumor surface to the deepest point of tumor invasion (3, 20, 21), was measured in the SEP tumors.

### Statistical Analyses

December 2017 was the data cut-off date for the analyses of disease-specific survival and overall survival. The median follow-up period for the 69 patients was 44 months (range, 2–144 months). Survival time was estimated using the Kaplan–Meier method. Comparisons of the CLNM-free survival between the groups were estimated using the log-rank test and Cox regression with Firth's penalized likelihood. Further, statistical evaluation of the associations between two categorical variables was performed using Fisher's exact test. All statistical tests were two-sided; a *p* < 0.05 was regarded as statistically significant. All statistical analyses were performed using R version 3.4.1 (R Foundation for Statistical Computing, Vienna, Austria) and EZR version 1.37 (Saitama Medical Center, Jichi Medical University, Saitama, Japan) (22).

## Results

### Patients' Clinical Data

Table 1 summarizes the demographic data of the patients. The median age was 70 years (range, 36–86 years), and 63 patients (91.3%) were men. The primary site was the hypopharynx in 57 lesions (82.6%), oropharynx in 11 lesions (15.9%), and larynx in 1 lesion (1.4%). No patients had synchronous SHNC. The incidence of synchronous and metachronous carcinoma was 18.8% (13/69) in the head and neck (not SHNC), 44.9% (31/69) in the esophagus, 13.0% (9/69) in the stomach, and 14.5% (10/69) in other areas, with overlaps.

**Table 1:** Patients' background.

Age (median, years)		36–86 (70)	
Sex (male:female)		63:6	
Primary tumor site			
Hypopharynx	Pyriform sinus	48	69.6%
	Postcricoid	2	2.9%
	Posterior wall	7	10.1%
Oropharynx		11	15.9%
Larynx		1	1.4%
Synchronous or metachronous cancer^*^			
Head and Neck		13	18.8%
Esophagus		31	44.9%
Gastric		9	13.0%
Other		10	14.5%

* *Data is duplicated*.

### Endoscopic and Pathological Findings

Table 2 shows the endoscopic and permanent pathological findings of SHNC. Type 0-IIb was the most common macroscopic type (45 patients, 65.2%). Type B1 was the most common microvessel irregularity (48 patients, 69.6%), followed by type B2 (16 patients, 23.2%) and type B3 (five patients, 7.2%). We identified AVA in 15 patients (21.7%). AVA was classified as small, middle, and large in eight (11.6%), five (7.2%), and two patients (2.9%), respectively (data not shown).

**Table 2:** Endoscopic and pathological findings.

**Macroscopic type (*N*,%)**		
0-Is	4	5.8%
0-IIa	13	18.8%
0-IIb	45	65.2%
0-IIc	7	10.1%
**Classification of type B vessels (*N*,%)**		
B1	48	69.6%
B2	16	23.2%
B3	5	7.2%
**AVA (*N*,%)**		
Present	15	21.7%
Absent	54	78.3%
**Pathological T stage (*N*,%)**		
Tis	37	53.6%
T1	7	10.1%
T2	17	24.6%
T3	8	11.6%
**Depth of invasion**		
EP	37	53.6%
SEP	32	46.4%
**Lymphatic invasion**		
Negative	67	97.1%
Positive	2	2.9%
**Venous invasion**		
Negative	68	98.6%
Positive	1	1.4%
**Surgical margin (*N*,%)**		
Negative	54	78.3%
Positive	12	17.4%
Undetermined	3	4.3%
Tumor width (mm)		
median (range)	23 (3–65)	
Tumor thickness (*N* = 32, mm)		
median (range)	1,225 (420–4,100)	

*AVA, avascular area; SEP, subepithelial propria; EP, epithelium*.

The pathological T stage was pTis in 37 patients (53.6%), pT1 in seven patients (10.1%), pT2 in 17 patients (24.6%), and pT3 in eight patients (11.6%). The depth of invasion was EP and SEP in 37 (53.6%) and 32 patients (46.4%), respectively. No tumors invaded the muscularis propria. Venous invasion was confirmed in only one patient (1.4%) while lymphatic invasion was confirmed in two patients (2.9%). The surgical margin was positive in 12 patients (17.4%) and indeterminable in 3 patients (4.3%). The median tumor width was 23 mm (3–65 mm), and the median thickness of SEP tumors was 1,225 (420–4,100) μm.

### Treatment Outcomes

Endoscopic submucosal dissection, endoscopic laryngopharyngeal surgery, and transoral videolaryngoscopic surgery were performed on 62 (89.9%), 5 (7.2%), and 2 patients (2.9%), respectively. The 5-year disease-specific and overall survival rates were 100% and 74.9%, respectively. Thirteen patients (18.8%) died of synchronous or metachronous cancer. No patient experienced local recurrence. However, 3 of 69 patients (4.3%) developed CLNM. The 5-year CLNM-free survival rate was 95.4%.

### Endoscopic and Pathological Findings and Treatment Outcomes of Three Cases With CLNM

**Figure 3:**
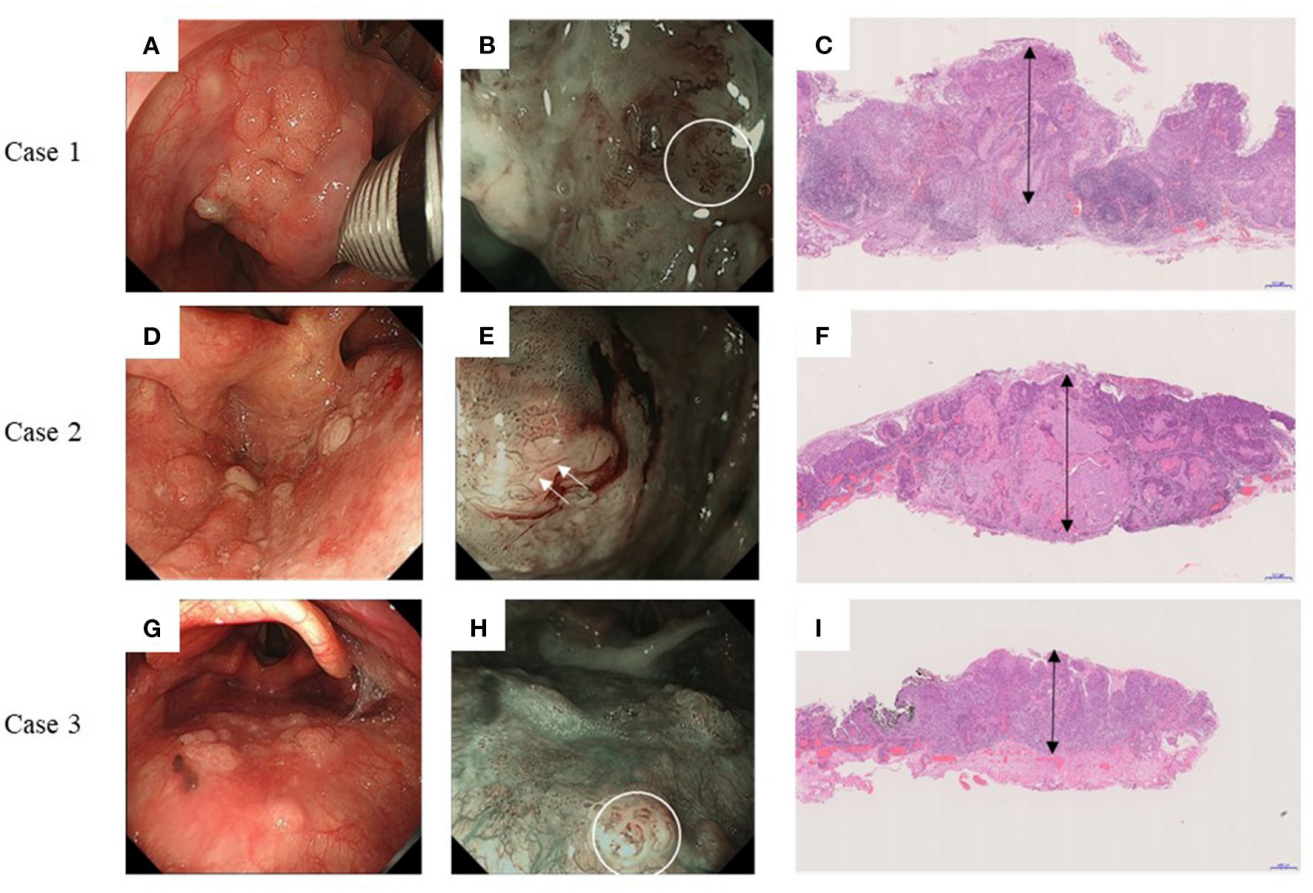
Endoscopic and pathological findings of three cases with delayed cervical lymph node metastasis. Left panels: Endoscopic findings under white right; Middle panels: Endoscopic findings under narrow-band imaging; and Right panels: Postoperative pathological findings. **(A–C)** Case 1. Type 0-IIa tumor on the left pyriform sinus. Type B2 vessels are indicated by white circles. The tumor thickness is 3,600 μm (double-headed arrow). **(D–F)** Case 2. Type 0-IIa tumor on the posterior wall of left pyriform sinus. Type B3 vessels are indicated by white arrows. The tumor thickness is 3,000 μm (double-headed arrow). **(G–I)** Case 3. Type 0-IIa tumor on the posterior wall with type B2 vessels indicated by white circles. The tumor thickness is 1,900 μm (double-headed arrow).

Figure 3 shows the preoperative endoscopic findings and postoperative pathological findings of all three patients with CLNM. Case 1 had a Type 0-IIa tumor on the left pyriform sinus (Figures 3A–C) with type B2 vessels, and tumor thickness was 3,600 μm (double-headed arrow). Case 2 had a Type 0-IIa tumor on the posterior wall of the left pyriform sinus with type B3 vessels (Figures 3D–F), and tumor thickness was 3,000 μm (double-headed arrow). Case 3 had a Type 0-IIa tumor on the posterior wall with observable type B2 vessels (Figures 3G–I), and tumor thickness was 1,900 μm (double-headed arrow).

All CLNMs occurred within a year of endoscopic resection, for which the patients underwent salvage therapy. We performed neck dissection followed by postoperative radiotherapy for two patients and concurrent chemoradiotherapy for one patient. All patients survived without recurrence or metastasis at the cut-off date.

### Predictive Factors for CLNM

The log-rank test and Cox regression revealed an association between CLNM and the IIa macroscopic type (hazards ratio [HR], 32.85), B2 or B3 type vessels (HR, 15.402), pT2 or pT3 stage (HR, 13.163), positive lymphatic invasion (HR, 97.5), positive surgical margin (HR, 27.71), and >1,000 μm tumor thickness (HR, 16.213) (Table 3). All three cases with CLNM had SEP invasion. As shown in Table 4, tumor thickness shows a significant correlation with the classification of type B vessels (*p* = 0.018).

**Table 3:** Log rank test and univariate Cox regression analyses of delayed lymph node metastasis-free survival.

	**Log rank test**	**Cox regression**		
	***p* value**	**HR**	**95%CI**	***p* value**
Age <70 vs. ≥70	0.516	2.173	0.197–23.97	0.527
location hypopharynx vs. others	0.405	1.619	0.157–217.752	0.735^†^
Macroscopic type Others vs. IIa	<0.001^*^	32.85	3.178–4419.686	0.002^*^^†^
Type B vessel B2/3 vs. B1	0.0102^*^	15.402	1.494–2070.895	0.019^*^^†^
AVA present vs. absent	0.374	1.843	0.179–247.830	0.663^†^
pathological tstage T2/3 vs. Tis/1	0.018^*^	13.163	1.274–v1770.938	0.028^*^^†^
Invasion SEP vs. EP	0.052	8.853	0.858–1190.693	0.07^†^
Lymphatic invasion present vs. absent	<0.001^*^	97.5	5.201–14227.23	0.004^*^^†^
Venous invasion present vs. absent	0.827	8.986	0.067–92.686	0.263^†^
Surgical margin positive vs. negative+indeterminable	<0.001^*^	27.71	2.508–306.1	0.007^*^^†^
Tumor thickness ≥1,000 vs. <1,000	0.008^*^	16.213	1.572–2180.189	0.017^*^^†^

*AVA, avascular area; SEP, subepithelial propria; EP, epithelium; HR, hazard ratio; CI, Confidence interval*.

* *Statistically significant*.

† *Firth's correction was used because of quasi-complete separation; there was no event in one of the subgroups*.

**Table 4:** Association between tumor thickness over 1,000 μm and clinicopathological factors.

	***p* value**
Age <70 vs. ≥70	1
Primary tumor site Hypopharynx vs. others	1
Macroscopic type Others vs. IIa	0.155
Type B vessel B2/3 vs. B1	0.0184^*^
AVA Present vs. absent	1
Pathological Tstage T2/3 vs. T1	0.648
Lymphatic invasion Present vs. absent	0.534
Venous invasion Present vs. absent	1
Surgical margin Positive vs. negative+indeterminable	1

*AVA, avascular area*.

* *Statistically significant*.

## Discussion

### Treatment Outcomes

Previous studies have reported 100% 5-year disease-specific survival rates in patients who have undergone endoscopic resection for SHNC (1, 10, 11). The incidence of local recurrence in SHNC is reportedly 1.2–13% (8–10). Consistent with these reports, none of our patients died from SHNC, and the 5-year disease-specific survival rate was 100%. Furthermore, there was no local recurrence. Different from disease-specific survivals, the 5-year overall survival rate was as low as 74.9% in this study. Thirteen patients died from synchronous or metachronous cancer in the upper aerodigestive tract known as “field cancerization” (23). It is suggested that endoscopic resection is a non-invasive and beneficial strategy for SHNC, while a careful exploration of metachronous as well as synchronous cancer is extremely important in patients with SHNC.

The incidence of synchronous and/or metachronous CLNM in SHNC is reportedly 2–19.4% (8–12). In the present study, CLNM after endoscopic resection occurred in 4.3% (3/69) of patients with SHNC and in 9.4% (3/32) of patients with SEP tumors. Despite being rare, CLNM can occur in SHNC following an endoscopic surgery, and its early detection would provide a less invasive salvage treatment for patients. A careful follow-up is particularly important for those at risk of CLNM following initial treatment. This, in turn, highlights the significance of identifying the predictive factors of CLNM following endoscopic surgery, which is discussed in the next paragraph.

### Predictive Factors for CLNM

In clinical practice, predicting CLNM after endoscopic resection is equally important to detecting synchronous CLNM, as the latter is usually involved in planning the initial treatment strategy while the former affects follow-up after the initial treatment. Therefore, we focused on the correlation of CLNM following endoscopic surgery with possible predictors among the clinicopathological factors. Statistical tests showed that endoscopic findings, including macroscopic IIa type and type B2/B3 vessels, were significantly associated with CLNM following endoscopic resection. As pathological findings, ≥pT2 stage tumor, lymphatic invasion, positive surgical margin, and tumor thickness of ≥1,000 μm in SEP tumors showed significant association with CLNM following endoscopic resection (Table 3). Based on these findings, screening of high-risk CLNM cases by these preoperative endoscopic findings followed by confirmation using the above postoperative pathological findings may be an ideal assessment for a strict follow-up for possible CLNM following endoscopic resections.

### Association Between Tumor Thickness and Classification of Type B Vessels

In superficial esophageal squamous cell carcinoma, the rate of nodal metastasis is highly dependent on the tumor depth (Figure 1); 0–9.1% in Tis (EP) or T1a tumor (LPM and MM) and 15.4–44.1% in T1b tumor (SM1–SM3) (Figure 1) (14). In order to predict the depth of the tumor, classification of type B vessels is particularly useful; type B1 vessels are significantly correlated with EP/LPM cancer, type B2 with MM/SM1, and type B3 with SM2/SM3 (17). Therefore, endoscopic surgeries are being performed for superficial esophageal squamous cell carcinoma showing type B1 or B2 vessels, which are expected to invade SM1 or less and have a lower risk of lymph node metastasis (Figure 1).

In contrast, because the pharyngeal mucosa lacks MM, the depth of the tumor has been classified into only two categories, i.e., EP and SEP in SHNC, so that EP/SEP may not be a sensitive correlate with CLNM (Table 3). As an alternative to the depth of invasion, several reports demonstrated that the tumor thickness correlated with CLNM, and tumor thickness of 1,000 μm was the threshold for risk of CLNM in SHNC (8, 9, 12). In these previous studies, however, statistical analyses of tumor thickness and CLNM were not fully conducted. Our results showed a statistically significant association between tumor thickness and CLNM (Table 3). As predictors for tumor thickness, Eguchi et al. have demonstrated a significant correlation between the classification of type B vessels and tumor thickness in superficial pharyngeal cancer (20). Katada et al. also have reported on the substantial correlation between the classification of type B vessels and synchronous lymph node metastasis/lymphatic invasion in superficial pharyngeal cancer (21). In the current study, among the parameters showing a significant correlation with CLNM, the classification of type B vessels was significantly associated with tumor thickness (Table 4). These results support the potential role of the classification of type B vessels in preoperative prediction for tumor thickness and risk of CLNM in SHNC, which may be useful for the selection of SHNC patients suitable for endoscopic resections plus watchful follow-up for possible CLNM.

A limitation of this study was the small number of CLNM-positive patients that were included; however, it should be noted that patients with SHNC rarely develop metachronous CLNM. Nonetheless, statistically significant predictors of CLNM may be obtained for comparison with non-CLNM cases as controls. Further prospective, large-scale observational studies are required to address the aforementioned drawbacks.

In conclusion, the treatment outcomes of SHNC following endoscopic surgery were favorable. However, CLNM following endoscopic resection was observed in 9.4% of patients with SEP tumors. Macroscopic type, classification of type B vessels under NBI, pathological T stage, lymphatic invasion, surgical margins, and tumor thickness were significantly associated with CLNM. Moreover, the classification of type B vessels had a significant correlation with tumor thickness as an alternative to the depth of invasion to assess the risk of CLNM. High-risk patients for CLNM can be predicted by the above preoperative endoscopic findings and postoperative pathological findings. Among them, the classification of type B vessels, which correlated with tumor thickness and CLNM, might be a useful preoperative measure for the selection of SHNC patients suitable for endoscopic resections.

## Data Availability Statement

The original data presented in the study are included in the article/supplementary material, further inquiries can be directed to the corresponding author/s.

## Ethics Statement

The studies involving human participants were reviewed and approved by the Institutional Review Board of the Niigata University Hospital (No. 2538). The patients/participants provided their written informed consent to participate in this study.

## Author Contributions

YU had complete access to the data and takes responsibility for its integrity, and the accuracy of the data analysis. ROk, YU, and AH: concept and design. ROk, YU, ROh, MT, SH, TT, RS, KY, HM, HU, ST, and YA: acquisition and analysis or interpretation of data. ROk, YU, ROh, and AH: drafting of the manuscript. ROk, YU, ROh: statistical analysis. YU, ROh, MT, SH, TT, RS, KY, and HM: administrative and technical or material support. AH: supervision. All authors: critical revision of the manuscript for important intellectual content.

## Funding

This study was supported by the Japan Society for Promotion of Sciences (JSPS) Grant in Aid for Scientific Research (C) #19K09884 to ROk.

## Conflict of Interest

The authors declare that the research was conducted in the absence of any commercial or financial relationships that could be construed as a potential conflict of interest.

## Publisher's Note

All claims expressed in this article are solely those of the authors and do not necessarily represent those of their affiliated organizations, or those of the publisher, the editors and the reviewers. Any product that may be evaluated in this article, or claim that may be made by its manufacturer, is not guaranteed or endorsed by the publisher.
